# COVID-19 related conspiracy beliefs and their relationship with defense strategies, emotions, powerlessness, attitudes, and time perspective

**DOI:** 10.3389/fpsyg.2022.939615

**Published:** 2022-10-11

**Authors:** Giovanna Celia, Giulia Lausi, Laura Girelli, Elisa Cavicchiolo, Pierpaolo Limone, Anna Maria Giannini, Mauro Cozzolino

**Affiliations:** ^1^Department of Humanities, Literature, Cultural Heritage, Education Sciences, University of Foggia, Foggia, Italy; ^2^Department of Psychology, Sapienza University of Rome, Rome, Italy; ^3^Department of Human, Philosophical and Educational Sciences, University of Salerno, Fisciano, Italy

**Keywords:** COVID-19 conspiratorial thinking, cluster analysis, defense mechanisms, coping strategies, time perspective

## Abstract

The COVID-19 pandemic has greatly impacted individual’s life and society, and such an emergency has increased the likelihood of recurring conspiratorial thinking. There is much research on broader conspiratorial thinking and studies on COVID-19-related conspiratorial thinking has been growing worldwide, moreover, the negative consequences of COVID-19 specific conspiratorial beliefs for people’s health are clear. However, person-centered research aiming at identify groups of individuals who share patterns of relations between COVID-19 specific conspiratorial beliefs and other psychological features is still scarce. A sample of 1.002 people (18–40 years old, *M* = 23; *SD* = 5.19) responded to a questionnaire administered online. The aim was to identify groups of individuals based on their beliefs about COVID-19 conspiracy theories and to compare the groups identified in terms of psychological characteristics associated such as automatic defense mechanisms, coping strategies, powerlessness, emotions, emotional regulation, attitudes toward the COVID-19, social distancing discontent, perceptions of COVID-19 severity and temporal perspective. A k-mean cluster analysis identified the groups of Believers (22.26%), Ambivalent believers (34.3%), and Non-believers (43.21%). The three groups differ particularly in terms of defense mechanisms, and time perspective. Results suggested the need to tailor interventions for individuals believing in COVID-19 conspiratorial theories based on differences in the psychological characteristics among the three groups.

## Introduction

The COVID-19 pandemic has greatly impacted individual’s life and society, imposing many limits on individual’s actions. In a time of serious and tragic social crisis, people look for ways to deal with fear, uncertainty, and lack of control, and, as research showed, this increases the likelihood of recurring to conspiratorial thinking (van Prooijen and Douglas, 2017) to provide a causal explanation to events to feel protected uncertainty and offers some compensatory sense of control (Douglas et al., 2017). Conspiracy theories can be described as “a subset of false beliefs in which the ultimate cause of an event is believed to be due to a plot by multiple actors working together with a clear goal in mind, often unlawfully and in secret” (Swami et al., 2014, pp. 220). It has been shown that conspiratorial beliefs have harmful consequences in the health domain. For example, among African-Americans, the conspiratorial beliefs that birth control and HIV/AIDS are forms of genocide against them were associated with negative attitudes toward contraceptive behaviors, which may have exposed people to unwanted pregnancies and sexually transmitted illnesses (Thorburn Bird and Bogart, 2003; Bogart and Thorburn, 2006; Hoyt et al., 2012). Moreover, it has been shown that endorsement of a variety of unrelated conspiracy theories is associated with negative attitudes toward vaccination (Jolley and Douglas, 2014; Lewandowsky et al., 2015). In the last 15 years many studies have investigated broader conspiratorial theories and found that they respond to at least three individual sorts of needs: epistemic needs, reflecting the desire to satisfy curiosity and to avoid uncertainty in understanding individuals’ environment; existential needs, as the desire to restore a threatened sense of security and control (see Kruglanski et al., 2021); social needs, including the desire to maintain a positive image of the self and the social group (see Douglas, 2021 for a review).

In the context of the actual emergency, COVID-19 conspiracy theories regarded the beliefs that COVID-19 is part of a government bioweapons program, that 5G cell towers are spreading COVID-19, or that pharmaceutical companies are encouraging the spread of COVID-19 for profit. Such beliefs are also associated with unhealthy or negative behaviors, for example supporting alternative and inefficacious remedies to fight COVID-19 as hydroxychloroquine (Bertin et al., 2020) or garlic and colloidal silver (Teovanović et al., 2021); it has also been seen that believing that 5G phone masts spread COVID-19 predicted intention to vandalize 5G masts and, more generally, to commit violence (Jolley and Paterson, 2020). Numerous conspiracy hypotheses may have been probably amplified by social media platforms which provide direct access to an unprecedented number of questionable contents (Cinelli et al., 2020; Rovetta and Bhagavathula, 2020; Rovetta and Castaldo, 2022).

Research has reported the negative consequences of COVID-19 specific conspiracy beliefs for people’s health: for instance, they were negatively associated with preventative behaviors such as wearing a mask, maintaining physical distancing, and willingness to vaccinate (Earnshaw et al., 2020; Allington et al., 2021). Studies also showed some psychological factors that could be associated with COVID-19-specific conspiratorial thinking, such as personality traits, emotions, lack of individual control, threat perception, perception of risk or mortality, and attitudes toward government actions (Biddlestone et al., 2020; Oleksy et al., 2021; Pellegrini et al., 2021).

Although many studies have investigated the factors and processes associated with both broader conspiratorial thinking and COVID-19-related conspiratorial thinking (variable-centered approaches), there are few person-centered research focused on identifying groups of individuals based on their levels of broader conspiratorial thinking, and, so far as we know, neither study identifies groups of individuals in terms of COVID-19-related conspiratorial thinking. For person-centered approach we mean studies that identify groups of individuals who share particular attributes or relations among attributes. Person-centered approaches are well suited for addressing questions that concern group differences in patterns, whereas variable-centered approaches describe associations between variables (Laursen and Hoff, 2006). Based on a person-centered approach, the first aim of the present study is to identify groups of individuals who share similar patterns in terms of conspiratorial beliefs about COVID-19 (e.g., the degree to which they think the virus was purposefully created in a lab in Wuhan or by pharmaceutical companies to sell their medications and vaccines) by means of a cluster analysis. The second aim was to compare the groups, identified by the cluster analysis, in terms of the psychological characteristics associated such as psychological defense mechanisms, coping styles, powerlessness, attitudes toward the norms, perception of coronavirus severity, social distancing discontent, and time perspective. As we stated above, although most of these factors were found to be associated with broader conspiratorial thinking or with specific COVID-19 conspiratorial beliefs, this has only been investigated through variables-center approaches. The present study aims to compare for the first time groups of individuals who share patterns of relations between conspiratorial beliefs about COVID-19 and psychological characteristics. Understanding these patterns plays a crucial role in identifying treatment targets and assigning appropriate interventions to people. In the next sections, we will outline the psychological factors we have considered in our study and provide a basis for their inclusion consistently with previous work on COVID-19-related or broader conspiracy beliefs.

## COVID-19 -related conspiracy beliefs and defense mechanisms

Defense mechanisms are automatic psychological processes that mediate an individual’s reaction to emotional conflict and internal and external stressors (American Psychiatric Association [APA], 2013). Usually operating without individual awareness, they are not a definite attempt to solve an issue, but a mental process to minimize feelings of anxiety. They can be classified in a hierarchy ranging from high adaptive to less adaptive levels (Thobaben, 2005; Metzger, 2014). The high-adaptive levels of defense mechanisms result in optimal adaptation in the handling of stressors and usually maximize feelings of well-being (American Psychiatric Association [APA], 2013). The defense mechanisms grouped in this level allow for the conscious awareness of feelings, ideas, and their consequences (American Psychiatric Association [APA], 2013). Humor and anticipation are two examples. An individual uses the defense mechanism of humor when he deals with internal/external stressors by emphasizing amusing and ironic aspects. Anticipation involves dealing with stress by experiencing or anticipating consequences and emotional reactions in advance and considering realistic alternative responses or solutions (American Psychiatric Association [APA], 2013). At a lower level of adaptiveness, dissociation is one other common defense level we refer to when an individual separates from reality by a temporary alteration in consciousness or identity (Thobaben, 2005). At an even lower level of adaptiveness, two examples of defense functioning are projection and denial (gross impairment in reality testing). They are characterized by the failure of defensive regulation to contain the individual’s reaction to stressors, leading to a pronounced break with objective reality (American Psychiatric Association [APA], 2013). An individual uses denial when unconsciously refuses to admit the existence of an unpleasant reality that is readily apparent to others, whereas he uses projection when unconsciously attributes unacceptable thoughts, feelings, or actions to another (e.g., blaming) (Thobaben, 2005). Finally, one example of defense mechanisms at the lowest level of adaptiveness is acting out. It is characterized by the use of physical actions instead of dealing with challenges directly by reflecting on and discussing feelings (Thobaben, 2005). Although it appears that there is no evidence so far of associations with COVID-19 specific conspiratorial beliefs, some studies showed that broader conspiracy theories were associated with psychological defense mechanisms (Albarracín, 2020), therefore, we found promising to include these factors in the comparison between the groups identified in terms of COVID-19 conspiratorial beliefs.

## COVID-19 -related conspiracy beliefs and coping

Coping is defined as “the cognitive and behavioral efforts made to master, tolerate, or reduce external and internal demands and conflicts among them” (Folkman and Lazarus, 1980). While psychological defense mechanisms are considered partially or largely unconscious and automatic, literature on coping emphasizes the conscious and volitional aim and behavioral and cognitive methods to attain those aims (Skinner et al., 2003), that represent possible malleable factors (Alivernini et al., 2019). Studies have shown that maladaptive coping strategies for example pseudo-epistemic coping (Swami et al., 2016), or avoidance coping strategies (Grant et al., 2013) could be related to broader conspiratorial thinking. Therefore, coping seems a potential factor to include when it comes to comparing the groups identified in terms of conspiratorial beliefs about COVID-19.

## COVID-19 -related conspiracy beliefs and emotions

As highlighted by Šrol et al. (2021), people’s emotional responses to the COVID-19 pandemic are crucial for understanding related conspiracy beliefs. People are more likely to believe in broader conspiracy theories when they experience negative emotions (Grzesiak-Feldman, 2013; Freeman and Bentall, 2017). Limited studies showed associations with some emotions, such as fear, anxiety, and worry, with COVID-19-related conspiracy beliefs. In particular, higher levels of anxiety, fear and worry about COVID-19 were associated with the belief that the disease is part of a conspiracy (Jovančević and Milićević, 2020; Sallam et al., 2020; Bruder and Kunert, 2021; Pellegrini et al., 2021). However, no studies so far have compared group of individuals who share patterns of relations between conspiratorial beliefs about COVID-19 and emotions (e.g., worry, surprise, fear, hope) or emotional regulations.

## COVID-19-related conspiracy beliefs and powerlessness

Another factor associated with conspiratorial beliefs relates to the capacity of such beliefs to make people feel more able to have a meaningful impact on important issues, an ability that is greatly under threat in such a pandemic crisis. Powerlessness – the perception of individuals about their incapacity to have an impact on relevant issues (Xiang et al., 2019) – was found to be associated both with general conspiracy theories (Douglas et al., 2017) and with conspiratorial thinking specific on COVID-19 (Biddlestone et al., 2020). However, so far, no studies have analyzed powerlessness through a person-centered approach in order to compare different groups of individuals identified in terms of conspiratorial beliefs about COVID-19.

## COVID-19 -related conspiracy beliefs: Associations with attitudes, perception of coronavirus severity, and social distancing discontent

Studies have also shown that COVID-19-related conspiratorial beliefs were associated with more negative attitudes toward government responses, perception of coronavirus severity, and discontent toward preventative measures (Georgiou et al., 2020; Pellegrini et al., 2021). However, no studies so far have investigated these factors in relations to conspiratorial beliefs about COVID-19 through a person-centered approach.

## COVID-19 -related conspiracy beliefs and time perspective

Many studies suggest that the way we view our past, present, and future affected a multitude of our daily behaviors. The typical way in which individuals segment the flow of their personal experiences into time categories can be used to describe individual differences (Zimbardo and Boyd, 2015). The researchers empirically distinguished five dimensions to describe individual time perspectives. The *Past-Negative* dimension reflects a generally negative, pessimistic, and regretful view of the past. The *Past Positive* refers to a nostalgic, warm, and sentimental attitude toward the past. The *Present Hedonistic* is characterized by an orientation toward present enjoyment and excitement ignoring future consequences. The *Present Fatalistic* factor reveals a belief in predestined future, the importance of fate, and a lack of control over life events. *Future* refers to a general future orientation characterized by planning for future aims and prospects of achievements (Zimbardo and Boyd, 2015). According to Zimbardo and Boyd (2015), time perspective allows flexible transition among the temporal orientations in particular situations, however, a specific orientation can be dominant for an individual. Especially present-oriented individuals may be best able to enjoy the moment as they would not be distracted by past worries, but they also may not be able to delay gratification. Individuals with high future orientation usually are good at setting and achieving goals and restraining themselves from engaging in risky behaviors. On the other hand, their ambitions may lead to neglect of personal and social relationships. Future orientation has been seen as related to several positive consequences for individuals, such as higher socioeconomic status, superior academic achievement, and fewer risk-taking behaviors (Fraisse, 1957; de Volder and Lens, 1982; Nuttin, 1985; Strathman et al., 1994; Zaleski, 1994; Levine, 1997; Petruccelli et al., 2014). According to Zimbardo and Boyd (2015), people-oriented toward the Present Fatalistic feel their lives dominated by external forces rather than by their behaviors, whereas Future-oriented people are supposed to be more self-responsible, look more after their health, and to seek long-term gratification. From the argument exposed above, we found time perspective a promising factor to include when it comes to comparing the groups identified in terms of conspiratorial beliefs about COVID-19.

## The current study

The present study contributes to this research area by identifying meaningful groups of individuals based on their conspiratorial beliefs about COVID-19. Moreover, the groups identified will be compared in terms of psychological characteristics, related to conspiratorial thinking. Consequently, two main research questions (RQ) guide this study: RQ1: Which groups of individuals are identified in terms of COVID-19 conspiratorial beliefs? RQ2: Are there any differences between the groups identified in terms of their psychological characteristics?

## Materials and methods

### Participants

Data were collected from December 2020 to January 2021, through an online questionnaire distributed throughout Italian universities. The questionnaire was spread through the Qualtrics platform (Qualtrics.XM^1^), including the requirement to fill in all questions in order not to have missing data. Participants could refuse their consent to fill in the questionnaire and drop out at any time. This study was conducted by the ethical standards of the Helsinki Declaration and was approved by the Institutional Review Board of the Department of Psychology of “Sapienza” University of Rome, with the following title: Psychological aspects of individual concerns and attitudes against Coronavirus in university students and prot. N. 0000305.

One thousand and ninety-nine questionnaires were collected; 1002 participants joined the research and accepted informed consent, while 87 refused (91.2% retention); 75.7% were female (N = 759); the age ranged from 18-to-40 years (*M* = 23; *SD* = 5.19). Further characteristics of the participants are shown in Table 1.

**TABLE 1:** Descriptive statistics of the sample.

		N	%
Gender	Male	243	24.25%
	Female	759	75.75%
Marital status	Not married	834	83.23%
	Married or in a registered partnership	154	15.37%
	Divorced	5	0.50%
	Separated	4	0.40%
	Widowed	5	0.50%
Geographic area	North-Italy	15	1.50%
	Center-Italy	157	15.67%
	South-Italy	830	82.83%

### Materials

For the present study, an online questionnaire consisting of different sections was developed; first, a summary of demographic data (i.e., age, gender, living conditions); then, the questionnaire included the measures presented below:

#### COVID-19 conspiracy scale

This 10-item scale (Biddlestone et al., 2020) was designed to measure beliefs in COVID-19 conspiracy theories (e.g., “Much information about the Coronavirus is deliberately kept from the public”); items are assessed on a 7-point Likert scale, ranging from “Strongly disagree” (1) to “Strongly agree” (7). Scale reliability was strong (Cronbach’s alpha = 0.906; S.E. = 2.28).

#### The reduced version of “The Defense Style Questionnaire 40”

This scale, through 14 items (e.g., “Often, when I am annoyed by something, I react impulsively”), aims to measure the level of defense mechanisms (Bovey and Hede, 2001); items are assessed on a 7-point Likert scale, ranging from “Strongly disagree” (1) to “Strongly agree” (7). The reliability of this scale was 0.671 (S.E. = 5.52), and subscales’ reliability ranged from 0.482 (S.E. = 1.72) to 0.724 (S.E. = 1.57).

#### The brief approach/avoidance coping questionnaire

This instrument assesses the “approach-avoidance” dichotomy in coping style through 12 items (e.g., “I am actively engaged in finding a solution to my problems”) on a 5-point Likert scale, ranging from “Disagree completely” (1) to “Agree completely” (5) (Finset et al., 2002); reliability of this scale was good in both factors, in accordance with the original study (Approach Cronbach’s alpha = 0.536; S.E. 2.37; Avoidance Cronbach’s alpha = 0.614; S.E. = 2.44).

#### The difficulties in emotion-regulation strategies

This scale measures individual difficulties in engaging in emotional regulation strategies through factors such as the recognition of one’s own emotions, the acceptance of negative emotions, the ability to regulate one’s own emotions, and to direct one’s behavior toward goals despite negative emotions (Lausi et al., 2020); it is a self-reported questionnaire, consisting in 20 items (e.g., “I pay attention to how I feel”) on a 5-point Likert scale, running from “Almost never” (1) to “Almost always” (5). Subscales’ reliability ranged from 0.741 (Awareness; S.E. = 1.70) to 0.905 (Impulse; S.E. = 1.26).

#### The positive and negative affect schedule – shortened version

This scale is a self-reported that measures positive and negative affect through 10-items (5-item Positive Affect and 5-item Negative Affect); participants are asked to rate adjectives of varying mood states (e.g., “happy,” “cheerful,” “worried,” “sad”), based on how often they have felt that way during the past week; on a 5-point Likert scale ranging from “Never” (1) to “Always” (5) (Ebesutani et al., 2012). The scale has also been used in Italian subjects (Cozzolino et al., 2021). Scale’s reliability was good in both factors (Positive Affect Cronbach’s alpha = 0.800; S.E. = 1.74; Negative Affect Cronbach’s alpha = 0.786; S.E. = 1.84).

### Powerlessness

A three-item scale (e.g., “I feel that the Coronavirus is too big a problem for my actions to have an impact”) has been used in order to measure the sense of being unable to make a meaningful impact on important issues (Biddlestone et al., 2020); the responses are on a 5-point Likert Scale, ranging from “Strongly disagree” (1) to “Strongly agree” (5). Scale’s reliability was good (Cronbach’s alpha = 0.738; S.E. = 1.32).

#### The attitudes and moods about the new coronavirus

This scale aimed to investigate the attitudes and moods of respondents about the new Coronavirus (Mari et al., 2020). The items on the scale (e.g., “The Coronavirus is a mysterious and highly lethal virus capable of decimating the world’s population”) were evaluated on a 5-point Likert scale, from “Completely disagree” (1) to “Completely agree” (5). The reliability of this scale ranged from 0.449 (Negative Attitudes; S.E. = 1.04) to 0.792 (Positive Attitudes; S.E. = 1.55).

#### Social distancing discontent

Participants were asked to indicate on a 5-point Likert-scale, ranging from “Not at all” (1) to “Completely” (5) how discontent they were with following the rules of virus containment (Burrai et al., 2021); items were built following the World Health Organization recommendations (e.g., How unhappy are you with the recommendation of: “Reducing outside activities”, “Having to maintain a physically safe distance from people you meet”) and adapted from previous studies conducted in Italy (Mallia et al., 2020; Alivernini et al., 2021; Cavicchiolo et al., 2021); the scale’s reliability was good (Cronbach’s alpha = 0.770; S.E. = 1.62).

#### Perception of coronavirus severity

This set of six items investigates beliefs related to Coronavirus, including the likelihood of contracting the virus; contagiousness and severity of the virus; and concern related to the possibility of contracting the virus (Burrai et al., 2020). Items are assessed on a 5-point Likert scale, ranging from “Not at all” (1) to “Completely” (5).

#### Stanford time perspective inventory

This instrument provides a simple way to measure multiple temporal perspectives of individuals and it is built on a theoretical basis that examines the emotional, social, cognitive, and motivational processes that are supposed to contribute to, and are in turn influenced by, the functioning of the temporal perspective (e.g., “Every morning one should make a plan of the day”; D’Alessio et al., 2003); all the answers were 5-point response scales ranging from “Never” (0) to “Very often” (4). Subscales’ reliability ranged from 0.295 (Hedonistic Present; S.E. = 3.08) to 0.736 (Future; S.E. = 2.63).

### Data analysis

One thousand and two questionnaires were collected from students attending Italian universities. Statistical analyses were performed using IBM SPSS (Statistical Package for Social Sciences), Version 25.0 (IBM Corp. Released, 2021). The socio-demographic characteristics of the sample were investigated; the normality of the data distribution for each variable was investigated through Q-Q Plots; no non-normal data were found; values of internal consistency (Cronbach’s alpha) were calculated for each factorial scale and the total (Taber, 2018). Zero-order correlations using Pearson coefficient were computed among all the variables of the study. Moreover, a Linear Regression Analysis was conducted to investigate which variables best predict COVID-19 conspiracy scale scores; the stepwise method was selected with Confidence Intervals at 95%. COVID-19 conspiracy scale was selected as a dependent variable and variables showing a significant correlation with the COVID-19 conspiracy scale were selected as predictors. The standardized values of the scales showing significant correlations were used to perform a k-mean cluster analysis (Quick Cluster in SPSS; Steinley and Brusco, 2008) with the aim of identifying groups of individuals based on the labeling variable “COVID-19 conspiracy scale.” As we do not know of any study on this kind in the field of conspiratorial thinking, an explorative approach was adopted: it was determined *a priori* that iteration of the data should lead to three final clusters, according to the selection criteria leading to a sufficiently large number of people in each cluster (Milligan and Cooper, 1985; Kodinariya and Makwana, 2013). Iteration among the scale scores led to the identification of the three cluster profiles based on the proximity of the scores to the centroids of the clusters themselves. The mean score of the labeling variable (i.e., COVID-19 conspiracy scale) was used to name the three clusters: a group with higher scores (i.e., COVID-conspiracy believers), a group with the average scores (COVID-conspiracy ambivalent believers), and a group with the lowest scores (COVID-conspiracy non-believers). The resulting clusters were then compared with each other in terms of psychological characteristics by performing ANOVAs using Bonferroni’s *post hoc* analysis with significance at *p* < 0.001.

## Results

### Correlation analysis

Pearson’s *r* correlation analyses (Table 2) showed both positive and negative scores between Conspiracy scores and several of the examined subscales. The correlation effect size ranged from small to medium. Only low significant correlations (Greenland et al., 2016) were found between Conspiracy scores and STPI subscale “Hedonistic Present” (*p* = 0.299); DSQ subscales “Anticipation” (*p* = 0.137) and “Isolation” (*p* = 0.931); BACQ “Approach” (*p* = 0.064) subscale; DERS “Goals” (*p* = 0.626), “Clarity” (*p* = 0.392) and “Awareness” (*p* = 0.204) subscales and PANAS negative affect (*p* = 0.851) (See Supplementary Tables 1, 2 for total correlations and their confidence intervals).

**TABLE 2:** Correlation analysis among the COVID-19 conspiracy scale and the examined subscales.

Variable	CONSP	DSQ_ AO	DSQ_ H	DSQ_ DEN	DSQ_ DIS	DSQ_ P	BACQ_ AV	DERS_ NA	DERS_ I	POS_ A	POW	AMAC_ P	AMAC_ N	DISC	COVID_ S	COVID_ C	COVID_ W	FUTURE	FATALIST
CONSP	−																		
DSQ_AO	0.082**	−																	
DSQ_H	−0.130**	–0.012	−																
DSQ_DEN	0.137**	0.090**	0.137**	−															
DSQ_DIS	0.164**	–0.017	0.152**	0.520**	−														
DSQ_P	0.118**	0.264**	−0.107**	0.152**	0.003	−													
BACQ_AV	0.107**	0.194**	−0.092**	0.167**	0.037	0.374**	−												
DERS_NA	0.083**	0.194**	–0.024	0.112**	–0.035	0.350**	0.336**	−											
DERS_I	0.078**	0.485**	–0.049	0.151**	0.011	0.345**	0.268**	0.493**	−										
POS_A	0.063*	–0.025	0.174**	0.116**	0.248**	−0.233**	−0.177**	−0.165**	−0.089**	−									
POW	0.259**	0.119**	–0.039	0.134**	0.087**	0.221**	0.186**	0.121**	0.122**	−0.099**	−								
AMAC_P	−0.150**	0.028	–0.033	−0.138**	−0.122**	−0.091**	0.018	–0.001	−0.060*	0.048	−0.131**	−							
AMAC_N	0.429**	0.016	–0.009	0.167**	0.134**	0.108**	0.050	0.095**	0.106**	0.016	0.245**	−0.411**	−						
DISC	0.137**	0.105**	0.009	–0.028	−0.138**	0.065*	0.073*	0.143**	0.096**	−0.107**	0.109**	0.010	0.088**	−					
COVID_S	−0.251**	–0.001	–0.012	−0.124**	−0.150**	–0.057	−0.070*	0.000	–0.031	–0.016	−0.179**	0.296**	−0.376**	–0.024	−				
COVID_C	−0.180**	0.011	–0.002	−0.076*	−0.123**	–0.040	0.013	0.017	0.008	–0.045	−0.069*	0.309**	−0.313**	0.044	0.384**	−			
COVID_W	−0.096**	0.005	−0.089**	−0.202**	−0.217**	–0.037	0.004	0.015	–0.012	–0.030	−0.106**	0.321**	−0.304**	0.071*	0.344**	0.294**	−		
FUTURE	0.111**	−0.122**	0.011	−0.077*	0.041	−0.209**	−0.176**	−0.070*	−0.139**	0.178**	−0.114**	0.136**	0.016	–0.002	0.099**	0.056	0.075*	−	
FATALIST	0.200**	0.466**	–0.009	0.113**	0.032	0.196**	0.259**	0.181**	0.358**	–0.001	0.174**	0.039	0.161**	0.138**	−0.070*	0.003	–0.010	−0.286**	-

CONSP, COVID-19 Conspiracy Scale; DSQ_AO, Acting Out subscale of DSQ; DSQ_H, Humor subscale of DSQ; DSQ_DEN, Denial subscale of DSQ; DSQ_DIS, Dissociation subscale of DSQ; DSQ_P, Projection subscale of DSQ; BACQ_AV, Avoidance subscale of BACQ; DERS_NA, Non-Acceptance subscale of DERS-20; DERS_I, Impulse subscale of DERS-20; POS_A, Positive Affect subscale of PANAS; POW, Powerlessness; AMAC_P, Positive Attitudes and Mood toward Coronavirus; AMAC_N, Negative Attitudes and Mood toward Coronavirus; DISC, Discontent for COVID-19; COVID_S, Beliefs toward Coronavirus Severity; COVID_ C, Beliefs toward Coronavirus Contagiousness; COVID_ W, Worrying for Coronavirus; FUTURE, Future subscale of STPI; FATALIST, Fatalistic Present subscale of STPI.

*, Correlation is significant at the 0.05 level (2-tailed).

**, Correlation is significant at the 0.01 level (2-tailed).

### Regression analysis

A stepwise regression analysis was conducted to identify whether COVID-19 Conspiracy scale could be predicted by any of the variables which showed statistically significant correlations; seven models were found (Table 3), with an R Square ranging from 0.184 to 0.283. The following subscales were excluded: Acting Out, Denial and Projection subscales of DSQ; Avoidance BACQ subscale, Non-Acceptance and Impulse subscales of DERS-20, both PANAS subscales, Positive Attitudes and Moods toward Coronavirus, beliefs toward coronavirus contagiousness, severity and worrying.

**TABLE 3:** Stepwise regression analysis among the COVID-19 conspiracy scale and the examined subscales.

Model		Unstandardized coefficients		Standardized coefficients	t	Sig.	95.0% Confidence interval for B		Change statistics			R Square
								
		B	Std. error	Beta			Lower bound	Upper bound	F	df1	df2	
1	(Constant)	10.818	0.630		17.170	0.000	9.581	12.054				
	AMAC_N	0.943	0.060	0.429	15.749	0.000	0.825	1.060	248.038	1	1099	0.184
2	(Constant)	8.466	0.737		11.482	0.000	7.019	9.912				
	AMAC_N	0.855	0.061	0.389	14.053	0.000	0.735	0.974				
	POW	0.473	0.080	0.164	5.907	0.000	0.316	0.630	32.894			0.209
3	(Constant)	2.157	1.530		1.410	0.159	–0.845	5.160				
	AMAC_N	0.842	0.060	0.383	13.957	0.000	0.723	0.960				
	POW	0.518	0.080	0.179	6.489	0.000	0.362	0.675				
	FUTURE	0.183	0.039	0.126	4.692	0.000	0.106	0.259	22.014	1	1098	0.225
4	(Constant)	–4.440	1.885		–2.355	0.019	–8.139	–0.741				
	AMAC_N	0.792	0.060	0.361	13.198	0.000	0.675	0.910				
	POW	0.468	0.079	0.162	5.903	0.000	0.312	0.623				
	FUTURE	0.248	0.040	0.171	6.205	0.000	0.170	0.327				
	FATALIST	0.358	0.061	0.163	5.829	0.000	0.238	0.479	33.973	1	1097	0.248
5	(Constant)	–0.830	2.023		–0.410	0.682	–4.800	3.140				
	AMAC_N	0.792	0.059	0.361	13.323	0.000	0.676	0.909				
	POW	0.454	0.079	0.157	5.783	0.000	0.300	0.608				
	FUTURE	0.249	0.040	0.171	6.292	0.000	0.172	0.327				
	FATALIST	0.358	0.061	0.163	5.879	0.000	0.239	0.478				
	DSQ_H	–0.341	0.073	–0.121	–4.641	0.000	–0.485	–0.197	21.538	1	1096	0.263
6	(Constant)	–1.204	2.009		–0.600	0.549	–5.146	2.737				
	AMAC_N	0.764	0.059	0.348	12.861	0.000	0.647	0.880				
	POW	0.432	0.078	0.149	5.532	0.000	0.279	0.585				
	FUTURE	0.241	0.039	0.166	6.124	0.000	0.164	0.318				
	FATALIST	0.354	0.060	0.161	5.850	0.000	0.235	0.472				
	DSQ_H	–0.391	0.074	–0.138	–5.301	0.000	–0.536	–0.246				
	DSQ_DIS	0.355	0.082	0.114	4.328	0.000	0.194	0.516	18.735	1	1094	0.275
7	(Constant)	–3.752	2.134		–1.758	0.079	–7.939	0.435				
	AMAC_N	0.751	0.059	0.342	12.677	0.000	0.634	0.867				
	POW	0.408	0.078	0.141	5.228	0.000	0.255	0.561				
	FUTURE	0.234	0.039	0.161	5.973	0.000	0.157	0.311				
	FATALIST	0.327	0.061	0.149	5.398	0.000	0.208	0.446				
	DSQ_H	–0.401	0.073	–0.142	–5.458	0.000	–0.545	–0.257				
	DSQ_DIS	0.402	0.083	0.129	4.855	0.000	0.240	0.565				
	DISC	0.199	0.058	0.090	3.414	0.001	0.085	0.314	11.6581	1	1093	0.283

Dependent Variable: COVID-19 Conspiracy Scale; AMAC_N, Negative Attitudes and Mood toward Coronavirus; POW, Powerlessness; FUTURE, Future subscale of STPI; FATALIST, Fatalistic Present subscale of STPI; DSQ_H, Humor subscale of DSQ; DSQ_DIS, Dissociation subscale of DSQ; DISC, Discontent for COVID-19.

### Cluster analysis

**FIGURE 1:**
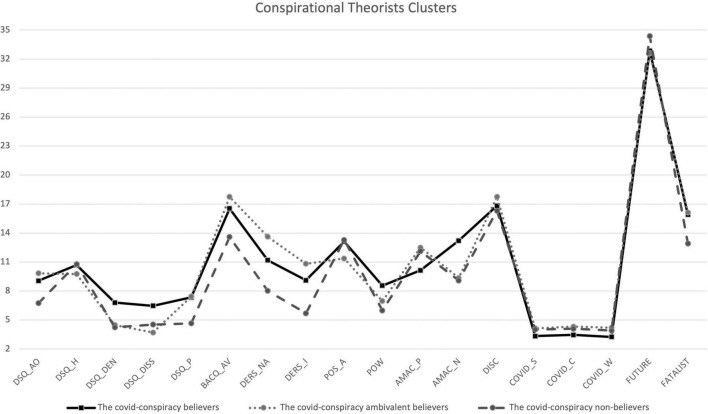
Graphical Representation of Clusters. DSQ_AO, Acting Out subscale of DSQ; DSQ_H, Humor subscale of DSQ; DSQ_DEN, Denial subscale of DSQ; DSQ_DISS, Dissociation subscale of DSQ; DSQ_P, Projection subscale of DSQ; BACQ_AV, Avoidance subscale of BACQ; DERS_NA, Non-Acceptance subscale of DERS-20; DERS_I, Impulse subscale of DERS-20; POS_A, Positive Affect subscale of PANAS; POW, Powerlessness; AMAC_P, Positive Attitudes and Mood toward Coronavirus; AMAC_N, Negative Attitudes and Mood toward Coronavirus; DISC, Discontent for COVID-19; COVID_S, Beliefs toward Coronavirus Severity; COVID_ C, Beliefs toward Coronavirus Contagiousness; COVID_ W, Worrying for Coronavirus; FUTURE, Future subscale of STPI; FATALIST, Fatalistic Present subscale of STPI.

The iteration and classification of standardized scores of the cluster analysis produced three groups of individuals in terms of conspiratorial beliefs about COVID-19, therefore they were labeled as (1) The COVID-conspiracy believers; (2) The COVID-conspiracy ambivalent believers; (3) The COVID-conspiracy non-believers. (See Figure 1 and Table 4) according to the scores of COVID-19 conspiracy scale.

**TABLE 4:** Sample clusters characterization.

Clusters	Cluster 1 (C1) COVID-conspiracy believers	Cluster 2 (C2) COVID-conspiracy ambivalent believers	Cluster 3 (C3) COVID-conspiracy non-believers
			
	N (%)	N (%)	N (%)
Sample size (%)	223 (22.26%)	346 (34.53%)	433 (43.21%)
	M (SD)	M (SD)	M (SD)
CONSP	26.32 (7.43)	19.64 (6.80)	17.40 (6.17)
DSQ_AO	9.06 (2.80)	9.84 (2.51)	6.74 (2.58)
DSQ_H	10.70 (2.40)	9.76 (3.04)	10.78 (2.37)
DSQ_DEN	6.80 (2.65)	4.49 (2.25)	4.25 (2.08)
DSQ_DISS	6.47 (2.70)	3.71 (2.00)	4.53 (2.04)
DSQ_P	7.35 (2.67)	7.43 (2.79)	4.66 (2.18)
BACQ_AV	16.54 (3.60)	17.75 (3.54)	13.59 (3.27)
DERS_NA	11.19 (4.66)	13.62 (5.56)	8.02 (3.46)
DERS_I	9.11 (3.93)	10.82 (4.39)	5.70 (2.04)
POS_A	13.13 (3.89)	11.37 (3.67)	13.29 (3.78)
POW	8.56 (2.51)	6.97 (2.52)	6.00 (2.18)
AMAC_P	10.14 (2.17)	12.50 (1.73)	12.12 (1.78)
AMAC_N	13.22 (2.77)	9.36 (3.07)	9.09 (2.97)
Disc	16.82 (3.52)	17.75 (3.27)	16.31 (3.16)
COVID-19 S	3.35 (0.69)	4.14 (0.64)	4.02 (0.63)
COVID-19 C	3.46 (0.73)	4.32 (0.62)	4.10 (0.73)
COVID-19 W	3.26 (0.88)	4.22 (0.62)	3.91 (0.68)
FUTURE	32.86 (5.17)	32.61 (5.20)	34.38 (4.85)
FATALIST	15.92 (2.98)	16.14 (3.10)	12.92 (2.90)

CONSP, COVID-19 Conspiracy Scale; DSQ_AO, Acting Out subscale of DSQ; DSQ_H, Humor subscale of DSQ; DSQ_DEN, Denial subscale of DSQ; DSQ_DISS, Dissociation subscale of DSQ; DSQ_P, Projection subscale of DSQ; BACQ_AV, Avoidance subscale of BACQ; DERS_NA, Non-Acceptance subscale of DERS-20; DERS_I, Impulse subscale of DERS-20; POS_A, Positive Affect subscale of PANAS; POW, Powerlessness; AMAC_P, Positive Attitudes and Mood toward Coronavirus; AMAC_N, Negative Attitudes and Mood toward Coronavirus; DISC, Discontent for COVID-19; COVID_S, Beliefs toward Coronavirus Severity; COVID_ C, Beliefs toward Coronavirus Contagiousness; COVID_ W, Worrying for Coronavirus; FUTURE, Future subscale of STPI; FATALIST, Fatalistic Present subscale of STPI.

The first cluster, named “COVID-conspiracy believers” represented the 22.26% of the sample, people belonging to this cluster showed the highest scores on the conspiracy scale and negative attitudes toward Coronavirus (AMAC_P *M* = 10.14, *SD* = 2.17; AMAC_N *M* = 13.22, *SD* = 2.77); this is also the group that most underestimated the severity and contagiousness of COVID-19. Regarding the sense of powerlessness, people belonging to this cluster showed the highest scores (*M* = 8.56, *SD* = 2.51) while regarding defense mechanisms, this is the cluster showing highest scores in the denial (*M* = 6.80, *SD* = 2.65) and dissociation (*M* = 6.47, *SD* = 2.70) subscales.

The second cluster was composed by 34.53% of the sample, named “COVID-conspiracy ambivalent believers”, showing middle scores on conspiracy scale. These participants showed the highest scores on the AMAC positive subscale (*M* = 12.50, *SD* = 1.73) but also on discontent due to social distancing norms (*M* = 17.75, *SD* = 3.27). It was also the cluster that perceived COVID-19 as more severe (COVID_S *M* = 4.14, *SD* = 0.64), contagious (COVID_C *M* = 4.32, *SD* = 0.62), and worrying (COVID_W *M* = 4.22, *SD* = 0.62) than seasonal flu. Within this group we found the highest scores on the DERS-20, both in the Non-Acceptance subscale (*M* = 13.62, *SD* = 5.56) and in the Impulse subscale (*M* = 10.82, *SD* = 4. 39); regarding defense mechanisms, members of this cluster seemed to use Acting Out (*M* = 9.84, *SD* = 2.51) and Projection (*M* = 7.43, *SD* = 2.79) strategies more; moreover, they showed the highest scores on the Avoidance subscale of the BACQ (*M* = 17.75, *SD* = 3.54), and lower scores on the PANAS Positive Affect subscale (*M* = 11.37, *SD* = 3.67). Regarding temporal perspective, this group showed the highest scores on the present fatalistic (*M* = 16.14, *SD* = 3.10).

Finally, the third group, labeled “COVID-conspiracy non-believers,” which shows the lowest mean scores on the conspiracy scale, was comprised of 43.21% of the sample. People within this cluster showed medium to low scores on the Coronavirus-related scales, compared to the other two clusters (Table 2), while showing the lowest scores on Emotional Dysregulation, Powerlessness and Avoidance Coping Strategies, and the highest scores on using humor as a defense mechanism (*M* = 10.78, *SD* = 2.37), the PANAS Positive Affect subscale (*M* = 13.29, *SD* = 3.78), and temporal orientation to the future (*M* = 34.38, *SD* = 4.85). Table 5 summarizes the significant results of the *post hoc* analyses performed among the groups to highlight the differences found among the three clusters and the sizes of the effects. Results showed a small effect for COVID-19 severity, humor subscale of DSQ, PANAS positive affect, and future subscale of STPI, while a large effect in the other dimensions.

**TABLE 5:** Differences between groups on COVID-related measures.

ANOVA										

	F	df	Sig.	Multiple comparisons	Mean difference	Std. error	Sig.	Eta-squared	90% Confidence Interval	
									
									Lower C.I.	Upper C.I.
CONSP	132.656	2.999	0.001	C1 vs. C2 C1 vs. C3 C2 vs. C3	6.68 8.92 2.23	0.574 0.551 0.481	0.001 0.001 0.001	0.209	0.173	0.244
AMAC_P	120.779	2.999	0.001	C1 vs. C2 C1 vs. C3	–2.37 –1.99	0.159 0.153	0.001 0.001	0.194	0.158	0.228
AMAC_N	160.391	2.999	0.001	C1 vs. C2 C1 vs. C3	3.87 4.14	0.254 0.244	0.001 0.001	0.243	0.205	0.278
DISC	18.492	2.999	0.001	C2 vs. C3	1.43	0.237	0.001	0.035	0.018	0.055
COVID-19 S	112.816	2.999	0.001	C1 vs. C2 C1 vs. C3	–0.79 –0.67	0.055 0.053	0.001 0.001	0.184	0.149	0.218
COVID-19 C	107.510	2.999	0.001	C1 vs. C2 C1 vs. C3 C2 vs. C3	–0.86 –0.64 0.22	0.060 0.057 0.050	0.001 0.001 0.001	0.177	0.142	0.210
COVID-19 W	125.678	2.999	0.001	C1 vs. C2 C1 vs. C3 C2 vs. C3	–0.96 –0.65 0.31	0.061 0.058 0.051	0.001 0.001 0.001	0.201	0.165	0.235
DERS_NA	148.078	2.999	0.001	C1 vs. C2 C1 vs. C3 C2 vs. C3	–2.44 3.17 5.61	0.391 0.375 0.328	0.001 0.001 0.001	0.228	0.191	0.263
DERS_I	220.798	2.999	0.001	C1 vs. C2 C1 vs. C3 C2 vs. C3	–1.71 3.40 5.11	0.296 0.284 0.249	0.001 0.001 0.001	0.306	0.268	0.341
POW	85.378	2.999	0.001	C1 vs. C2 C1 vs. C3 C2 vs. C3	1.59 2.56 0.96	0.204 0.196 0.171	0.001 0.001 0.001	0.145	0.113	0.178
DSQ_AO	146.878	2.999	0.001	C1 vs. C3 C2 vs. C3	2.32 3.09	0.215 0.188	0.001 0.001	0.227	0.190	0.262
DSQ_H	16.328	2.999	0.001	C1 vs. C2 C2 vs. C3	0.94 –1,02	0.226 0.189	0.001 0.001	0.031	0.015	0.050
DSQ_DEN	101.526	2.999	0.001	C1 vs. C2 C1 vs. C3	2.31 2.56	0.195 0.187	0.001 0.001	0.168	0.134	0.202
DSQ_DISS	108.749	2.999	0.001	C1 vs. C2 C1 vs. C3 C2 vs. C3	2.75 1.94 –0.82	0.188 0.180 0.158	0.001 0.001 0.001	0.178	0.143	0.212
DSQ_P	145.627	2.999	0.001	C1 vs. C3 C2 vs. C3	2.69 2.77	0.208 0.181	0.001 0.001	0.225	0.188	0.260
BACQ_AV	148.766	2.999	0.001	C1 vs. C2 C1 vs. C3 C2 vs. C3	–1.21 2.94 4.15	0.296 0.284 0.248	0.001 0.001 0.001	0.229	0.192	0.264
POS_A	27.883	2.999	0.001	C1 vs. C2 C2 vs. C3	1.76 –1.92	0.323 0.272	0.001 0.001	0.052	0.031	0.075
FATALIST	135.351	2.999	0.001	C1 vs. C3 C2 vs. C3	3.00 3.22	0.247 0.216	0.001 0.001	0.213	0.176	0.247
FUTURE	17.094	2.999	0.001	C1 vs. C3 C2 vs. C3	–2.03 –1.77	0.416 0.364	0.001 0.001	0.033	0.016	0.052

CONSP, COVID-19 Conspiracy Scale; DSQ_AO, Acting Out subscale of DSQ; DSQ_H, Humor subscale of DSQ; DSQ_DEN, Denial subscale of DSQ; DSQ_DISS, Dissociation subscale of DSQ; DSQ_P, Projection subscale of DSQ; BACQ_AV, Avoidance subscale of BACQ; DERS_NA, Non-Acceptance subscale of DERS-20; DERS_I, Impulse subscale of DERS-20; POS_A, Positive Affect subscale of PANAS; POW, Powerlessness; AMAC_P, Positive Attitudes and Mood toward Coronavirus; AMAC_N, Negative Attitudes and Mood toward Coronavirus; DISC, Discontent for COVID-19; COVID_S, Beliefs toward Coronavirus Severity; COVID_ C, Beliefs toward Coronavirus Contagiousness; COVID_ W, Worrying for Coronavirus; FUTURE, Future subscale of STPI; FATALIST, Fatalistic Present subscale of STPI. The mean difference is significant at the 0.001 level (*p* < 0.001); only significant comparisons are showed.

## Discussion

The COVID-19 pandemic has greatly impacted individual’s life and society and such emergency has increased the likelihood of recurring to conspiratorial thinking. Although several studies have identified the factors and processes associated to COVID-19 conspiratorial thinking, research on this topic, based on a person-centered approach, is still limited. Our first aim was to identify groups of individuals, based on conspiratorial beliefs about COVID-19, who share similar patterns; our second aim was to compare these groups in terms of psychological characteristics. Concerning our first aim, the cluster analysis identified three groups of individuals: the “COVID-conspiracy believers,” the “COVID-conspiracy ambivalent believers”, and the “COVID-conspiracy non-believers.” The “Believers” group, apart from showing the highest scores on the conspiracy scale, also showed the highest score on the negative attitude toward Coronavirus. This group also underestimated the most the severity and contagiousness of COVID-19 and showed the highest score on powerlessness, meant as the individual perception about their incapacity to have an impact on relevant issues. These results are in line with previous studies that found COVID-19-related conspiratorial beliefs associated with more sense of powerlessness and more negative attitudes toward COVID-19 (Georgiou et al., 2020; Pellegrini et al., 2021). This group also show highest scores in the use of defense mechanisms as “denial” (e.g., “People say I tend to ignore unpleasant facts as if they didn’t exist”) and “dissociation” (e.g., “I ignore danger as if I were Superman”) which have the goal to keeping unpleasant or unacceptable stressors, impulses, ideas, affects, or responsibility out of awareness. The “denial” represents the unconscious mechanism of disavowal that it is behind the willing suspension of disbelief and the temporarily abandon critical faculties. The “dissociation” allows to this kind of people to deal with internal/external stressors with a breakdown in the usually integrated functions of consciousness, memory, and perception of self or the environment. They also strongly use defense mechanisms such as “acting out” (e.g., “I get openly aggressive when I feel hurt”) and “projection” (e.g., “People tend to mistreat me”). The “acting out” is positioned at the lowest level of the hierarchy based on their level of adaptiveness (Metzger, 2014). Referring when “an individual deals with internal/external stressors by actions rather than reflections or feelings” (Bovey and Hede, 2001, p. 537). In support of this finding, the “Believers” group also seem to experience difficulties in engaging in emotional regulation strategies due to factors such as “high impulsivity” (e.g., “When I’m upset, I have difficulty controlling my behaviors”) and “low acceptance of negative emotions” (e.g., “When I’m upset, I become embarrassed for feeling that way”). “Projection” instead is a defense mechanism which involves our own unacceptable qualities or feelings and ascribing them to other people in order to reduce anxiety. For these reasons we can state that “Believers” tend to react to stress, anxiety, fear and worry by automatically and unconsciously activating a very archaic, primitive, and not very adaptive defense mechanisms pattern. This will have important effects on the way they perceive reality and/or others and it will orient them toward specific attitudes and behaviors that in some ways, even if they are dysfunctional and bizarre, are also very predictable. This extends the results of previous studies, who found associations between conspiracy theories with psychological defense mechanisms (Albarracín, 2020), to the COVID-19 specific conspiratorial beliefs.

Moreover, the Believers group has shown to be very oriented to present fatalistic, the so-called “What will be, will be” (e.g., “It doesn’t make sense to worry about the future since there is nothing to do about it anyway”). Based on this result, the Believers group think that everything is already determined by fate, that their lives are dominated by a predetermined plan over which they have little or no control (Zimbardo et al., 2012). Although no studies so far have examined the relationships between time perspective and COVID-19 conspiratorial thinking, according to the conceptualization of the fatalistic present, the “Believers” feel their lives dominated by external forces rather than by their behaviors.

The “Ambivalent believers” group showed intermediate scores on the conspiracy scale, and it was the group that perceived COVID-19 as more severe, contagious, and worrying than seasonal flu. These people experienced the highest scores in the positive attitudes and moods about the Coronavirus. On the other side, they also experienced the strongest discontent due to social distancing norms. Members of this group seem to use defense mechanisms such as “acting out” and “projection” which we know to be two types of defense mechanisms with a low level of adaptiveness (Metzger, 2014). They also use the avoidance coping strategy the most. As we have defined, they seem uncertain and ambivalent, almost indefinite, unlike “Believers” and “Non-believers” groups. The “Ambivalent believers” group showed the highest score in present fatalistic temporal orientation. Many studies evidenced that this kind of temporal perspective is correlated to low emotional well-being and high-risk level of feeling anxiety and depression (Zimbardo et al., 2012). For all that we can state that right, the “Ambivalent believers” group seem to show solid maladjustment to the COVID-19 issue compared to the “Believers” and the “Non-believers” group.

Finally, people within the “Non-believers” group (e.g., the cluster with the lowest scores on the conspiracy scale) showed medium to low scores on the Coronavirus-related scales, compared to the “Ambivalent believers” and the “Believers.” They showed the lowest scores on Emotional Dysregulation, Powerlessness, and Avoidance Coping Strategies evidencing better overall mind-body functioning which can also be noted from Intensive Positive Affectivity. Moreover, we found that they have the highest scores on using “humor,” known as one of the defense mechanisms at the highest levels of adaptiveness (Metzger, 2014).

Alongside this positive attitude to the difficult stage where the world is, they also have a very useful temporal perspective. They are more oriented toward the future than the “Believers” and the “Ambivalent believers.” As some other research showed, future-oriented people make decisions based on a reasoned evaluation of the consequences, they plan and are confident that their choices will work, and they tend to live longer thanks to their conscientiousness (Zimbardo et al., 2012).

Overall, the findings of the present study confirm the key role of emotions, attitudes, and powerlessness in COVID-19 conspiratorial thinking (Biddlestone et al., 2020; Jovančević and Milićević, 2020; Sallam et al., 2020; Bruder and Kunert, 2021; Pellegrini et al., 2021), and extend the results of previous studies who found defense mechanisms to be correlated with broader conspiratorial thinking to the COVID-19 specific conspiratorial beliefs (Albarracín, 2020), by using a person-centered approach focused on identifying groups of individuals based on their levels of conspiratorial thinking.

## Potential limitations and directions for future research

Some limitations of this study need to be considered. First, the number of clusters was chosen *a priori* using a selection method that considered solutions with a sufficiently large number of people within each group. Second, the majority of the participants in our sample were female, future studies should be conducted with a more balanced sample. Third, although this cluster analysis included some indicators of psychological well-being, future research could include other indicators of emotional aspects, particularly stress and anxiety (Rossi et al., 2011; Venuleo et al., 2018). Typologies based on valid psychometric measures of these critical aspects would be more informative for policy interventions. Finally, we should specify that our results are correlational in nature therefore caution should be taken in drawing causal inferences. Nonetheless, this study contributes to understanding COVID-19-related conspiracy beliefs, even considering these limitations. The study also provides empirical bases for developing more tailored programs and interventions to reduce COVID-19-related conspiracy beliefs prevalence and impacts by adopting a person-centered approach and identifying diverse and qualitative distinct groups of individuals based on specific psychological features.

## Conclusion

The COVID-19 pandemic has greatly impacted individuals’ lives and society (Bruno et al., 2021), imposing many limits on individuals’ actions that increased the likelihood of recurring conspiratorial thinking that has harmful consequences in the health domain. For instance, they were negatively associated with preventative behaviors such as wearing a mask, maintaining social distancing, and willingness to vaccinate. This is the first study conducted in this field through a person-centered approach, that identifies groups of individuals who share particular patterns in terms of conspiratorial beliefs about COVID-19 and psychological characteristics associated.

The study answered two different research questions: first, based on conspiratorial thinking about COVID-19, three different groups of individuals can be identified: “Believers,” “Ambivalent believers” and “Non-believers.” The second: the groups identified differ in terms of psychological characteristics, specifically in defense mechanisms, coping strategies, and temporal orientation. These results can be useful for structuring different prevention and communication paths concerning the specific profile.

## Data availability statement

The raw data supporting the conclusions of this article will be made available by the authors, without undue reservation.

## Ethics statement

The studies involving human participants were reviewed and approved by the Institutional Review Board of the Department of Psychology of “Sapienza” University of Rome (N. 0000305). The patients/participants provided their written informed consent to participate in this study.

## Author contributions

GC, MC, and AMG developed the theoretical framework of the present study, designed the study, and developed the methodological approach. GL, EC, and LG performed all the analyses and designed tables and figure. GC and LG led the literature search and interpretation of data. GC, MC, AMG, and PL critically revised the manuscript. MC contributed to the scientific supervision of the whole work. All authors made a substantial contribution to the work, read, and approved the final version of the work.

## Footnotes

1
https://www.qualtrics.com/
