# Supplementary material for: COVID-19 related conspiracy beliefs and their relationship with defense strategies, emotions, powerlessness, attitudes, and time perspective
Source: Front Psychol. 2022 Oct 11;13:939615. doi: 10.3389/fpsyg.2022.939615 (PMC9592837; doi:10.3389/fpsyg.2022.939615)
Supplement: Supplementary file 1 [file Table_1.DOCX]

| **Correlations** | | | | | | | | | | | | | | | |
| --- | --- | --- | --- | --- | --- | --- | --- | --- | --- | --- | --- | --- | --- | --- | --- |
|  | CONSP | DERS_NA | DERS_G | DERS_I | DERS_C | DERS_A | PSS | POS_A | NEG_A | BACQ_AP | BACQ_AV | DSQ_AO | DSQ_H | DSQ_AN | DSQ_I |
| CONSP | -- |  |  |  |  |  |  |  |  |  |  |  |  |  |  |
| DERS_NA | .083^**^ | -- |  |  |  |  |  |  |  |  |  |  |  |  |  |
| DERS_G | -.015 | .459^**^ | -- |  |  |  |  |  |  |  |  |  |  |  |  |
| DERS_I | .078^**^ | .493^**^ | .545^**^ | -- |  |  |  |  |  |  |  |  |  |  |  |
| DERS_C | .026 | .464^**^ | .358^**^ | .373^**^ | -- |  |  |  |  |  |  |  |  |  |  |
| DERS_AW | .038 | .166^**^ | .001 | .070^*^ | .295^**^ | -- |  |  |  |  |  |  |  |  |  |
| PSS | -.049 | .411^**^ | .523^**^ | .405^**^ | .412^**^ | .188^**^ | -- |  |  |  |  |  |  |  |  |
| POS_A | .063^*^ | -.165^**^ | -.233^**^ | -.089^**^ | -.230^**^ | -.128^**^ | -.386^**^ | -- |  |  |  |  |  |  |  |
| NEG_A | .006 | .359^**^ | .386^**^ | .325^**^ | .355^**^ | .124^**^ | .541^**^ | -.419^**^ | -- |  |  |  |  |  |  |
| BACQ_AP | .056 | -.162^**^ | -.167^**^ | -.146^**^ | -.269^**^ | -.378^**^ | -.317^**^ | .262^**^ | -.236^**^ | -- |  |  |  |  |  |
| BACQ_AV | .107^**^ | .336^**^ | .236^**^ | .268^**^ | .371^**^ | .194^**^ | .281^**^ | -.177^**^ | .279^**^ | -.202^**^ | -- |  |  |  |  |
| DSQ_AO | .082^**^ | .194^**^ | .301^**^ | .485^**^ | .236^**^ | .009 | .360^**^ | -.025 | .236^**^ | -.030 | .194^**^ | -- |  |  |  |
| DMQ_H | -.130^**^ | -.024 | -.024 | -.049 | -.050 | -.121^**^ | -.086^**^ | .174^**^ | -.072^*^ | .126^**^ | -.092^**^ | -.012 | -- |  |  |
| DMQ_AN | -.045 | -.080^**^ | -.106^**^ | -.089^**^ | -.110^**^ | -.180^**^ | -.206^**^ | .153^**^ | -.165^**^ | .255^**^ | -.145^**^ | -.041 | .303^**^ | -- |  |
| DMQ_I | -.003 | .198^**^ | .132^**^ | .180^**^ | .331^**^ | .219^**^ | .142^**^ | -.086^**^ | .120^**^ | -.255^**^ | .184^**^ | .119^**^ | .098^**^ | .086^**^ | -- |
| DMQ_DEN | .137^**^ | .112^**^ | -.006 | .151^**^ | .183^**^ | .118^**^ | -.077^*^ | .116^**^ | -.051 | -.097^**^ | .167^**^ | .090^**^ | .137^**^ | .083^**^ | .532^**^ |
| DMQ_DISS | .164^**^ | -.035 | -.153^**^ | .011 | .026 | .003 | -.308^**^ | .248^**^ | -.158^**^ | .103^**^ | .037 | -.017 | .152^**^ | .167^**^ | .186^**^ |
| DMQ_P | .118^**^ | .350^**^ | .314^**^ | .345^**^ | .415^**^ | .242^**^ | .465^**^ | -.233^**^ | .370^**^ | -.271^**^ | .374^**^ | .264^**^ | -.107^**^ | -.144^**^ | .230^**^ |
| DISC | .137^**^ | .143^**^ | .185^**^ | .096^**^ | .142^**^ | .018 | .170^**^ | -.107^**^ | .211^**^ | .047 | .073^*^ | .105^**^ | .009 | -.031 | .018 |
| POW | .259^**^ | .121^**^ | .097^**^ | .122^**^ | .169^**^ | .159^**^ | .133^**^ | -.099^**^ | .072^*^ | -.133^**^ | .186^**^ | .119^**^ | -.039 | -.052 | .146^**^ |
| AMAC_N | .429^**^ | .095^**^ | .050 | .106^**^ | .074^*^ | .025 | -.016 | .016 | -.016 | -.041 | .050 | .016 | -.009 | .005 | .066^*^ |
| AMACP | -.150^**^ | -.001 | .002 | -.060^*^ | -.027 | -.025 | .049 | .048 | .030 | .118^**^ | .018 | .028 | -.033 | .012 | -.078^**^ |
| FUTURE | .111^**^ | -.070^*^ | -.203^**^ | -.139^**^ | -.220^**^ | -.190^**^ | -.247^**^ | .178^**^ | -.135^**^ | .357^**^ | -.176^**^ | -.122^**^ | .011 | .320^**^ | -.149^**^ |
| HEDONIST | .031 | .101^**^ | .136^**^ | .203^**^ | .152^**^ | -.003 | .056 | .042 | .101^**^ | .018 | .125^**^ | .155^**^ | .127^**^ | .001 | .139^**^ |
| FATALIST | .200^**^ | .181^**^ | .241^**^ | .358^**^ | .242^**^ | .093^**^ | .216^**^ | -.001 | .159^**^ | -.103^**^ | .259^**^ | .466^**^ | -.009 | -.140^**^ | .084^**^ |
| COVID_S | -.251^**^ | .000 | -.004 | -.031 | -.026 | .011 | .070^*^ | -.016 | .051 | .001 | -.070^*^ | -.001 | -.012 | .018 | -.060^*^ |
| COVID_C | -.180^**^ | .017 | .016 | .008 | .007 | .010 | .072^*^ | -.045 | .048 | .004 | .013 | .011 | -.002 | .039 | .002 |
| COVID_W | -.096^**^ | .015 | .066^*^ | -.012 | -.003 | -.033 | .114^**^ | -.030 | .109^**^ | .008 | .004 | .005 | -.089^**^ | -.014 | -.086^**^ |

| **Correlations** | | | | | | | | | | | | | | |
| --- | --- | --- | --- | --- | --- | --- | --- | --- | --- | --- | --- | --- | --- | --- |
|  | DSQ_DEN | DSQ_DISS | DSQ_P | DISC | POW | AMAC_N | AMAC_P | FUTURE | HEDONIST | FATALIST | COVID-S | COVID-C | COVID-W |  |
| CONSP |  |  |  |  |  |  |  |  |  |  |  |  |  |  |
| DERS_NA |  |  |  |  |  |  |  |  |  |  |  |  |  |  |
| DERS_G |  |  |  |  |  |  |  |  |  |  |  |  |  |  |
| DERS_I |  |  |  |  |  |  |  |  |  |  |  |  |  |  |
| DERS_C |  |  |  |  |  |  |  |  |  |  |  |  |  |  |
| DERS_AW |  |  |  |  |  |  |  |  |  |  |  |  |  |  |
| PSS |  |  |  |  |  |  |  |  |  |  |  |  |  |  |
| POS_A |  |  |  |  |  |  |  |  |  |  |  |  |  |  |
| NEG_A |  |  |  |  |  |  |  |  |  |  |  |  |  |  |
| BACQ_AP |  |  |  |  |  |  |  |  |  |  |  |  |  |  |
| BACQ_AV |  |  |  |  |  |  |  |  |  |  |  |  |  |  |
| DSQ_AO |  |  |  |  |  |  |  |  |  |  |  |  |  |  |
| DSQ_H |  |  |  |  |  |  |  |  |  |  |  |  |  |  |
| DSQ_AN |  |  |  |  |  |  |  |  |  |  |  |  |  |  |
| DSQ_I |  |  |  |  |  |  |  |  |  |  |  |  |  |  |
| DSQ_DEN | -- |  |  |  |  |  |  |  |  |  |  |  |  |  |
| DSQ_DISS | .520^**^ | -- |  |  |  |  |  |  |  |  |  |  |  |  |
| DSQ_P | .152^**^ | .003 | -- |  |  |  |  |  |  |  |  |  |  |  |
| DISC | -.028 | -.138^**^ | .065^*^ | -- |  |  |  |  |  |  |  |  |  |  |
| POW | .134^**^ | .087^**^ | .221^**^ | .109^**^ | -- |  |  |  |  |  |  |  |  |  |
| AMAC_N | .167^**^ | .134^**^ | .108^**^ | .088^**^ | .245^**^ | -- |  |  |  |  |  |  |  |  |
| AMAC_P | -.138^**^ | -.122^**^ | -.091^**^ | .010 | -.131^**^ | -.411^**^ | -- |  |  |  |  |  |  |  |
| FUTURE | -.077^*^ | .041 | -.209^**^ | -.002 | -.114^**^ | .016 | .136^**^ | -- |  |  |  |  |  |  |
| HEDONIST | .189^**^ | .188^**^ | .124^**^ | .204^**^ | .138^**^ | .184^**^ | -.113^**^ | -.187^**^ | -- |  |  |  |  |  |
| FATALIST | .113^**^ | .032 | .196^**^ | .138^**^ | .174^**^ | .161^**^ | .039 | -.286^**^ | .298^**^ | -- |  |  |  |  |
| COVID_S | -.124^**^ | -.150^**^ | -.057 | -.024 | -.179^**^ | -.376^**^ | .296^**^ | .099^**^ | -.140^**^ | -.070^*^ | -- |  |  |  |
| COVID-C | -.076^*^ | -.123^**^ | -.040 | .044 | -.069^*^ | -.313^**^ | .309^**^ | .056 | -.109^**^ | .003 | .384^**^ | -- |  |  |
| COVID-W | -.202^**^ | -.217^**^ | -.037 | .071^*^ | -.106^**^ | -.304^**^ | .321^**^ | .075^*^ | -.123^**^ | -.010 | .344^**^ | .294^**^ | -- |  |

**. Correlation is significant at the 0.01 level (2-tailed). *. Correlation is significant at the 0.05 level (2-tailed).

| **Confidence Intervals** | | | | |
| --- | --- | --- | --- | --- |
|  | Pearson Correlation | Sig. (2-tailed) | 95% Confidence Intervals (2-tailed)^a^ | |
|  |  |  | Lower | Upper |
| CONSP - DERS_NA | .083 | .006 | .024 | .142 |
| CONSP - DERS_G | -.015 | .626 | -.074 | .044 |
| CONSP - DERS_I | .078 | .010 | .019 | .136 |
| CONSP - DERS_C | .026 | .392 | -.033 | .085 |
| CONSP - DERS_AW | .038 | .204 | -.021 | .097 |
| CONSP - PSS | -.049 | .105 | -.108 | .010 |
| CONSP - POS_A | .063 | .036 | .004 | .122 |
| CONSP - NEG_A | .006 | .851 | -.053 | .065 |
| CONSP - BACQ_AP | .056 | .064 | -.003 | .115 |
| CONSP - BACQ_AV | .107 | .000 | .048 | .165 |
| CONSP - DSQ_AO | .082 | .006 | .023 | .141 |
| CONSP - DSQ_H | -.130 | .000 | -.187 | -.071 |
| CONSP - DSQ_AN | -.045 | .137 | -.104 | .014 |
| CONSP - DSQ_I | -.003 | .931 | -.062 | .056 |
| CONSP - DSQ_DEN | .137 | .000 | .079 | .195 |
| CONSP - DSQ_DISS | .164 | .000 | .106 | .221 |
| CONSP - DSQ_P | .118 | .000 | .059 | .176 |
| CONSP - DISC | .137 | .000 | .078 | .194 |
| CONSP - POW | .259 | .000 | .203 | .313 |
| CONSP - AMAC_N | .429 | .000 | .379 | .476 |
| CONSP - AMAC_P | -.150 | .000 | -.207 | -.092 |
| CONSP - FUTURE | .111 | .000 | .053 | .169 |
| CONSP - HEDONIST | .031 | .299 | -.028 | .090 |
| CONSP - FATALIST | .200 | .000 | .142 | .256 |
| CONSP - COVID_S | -.251 | .000 | -.305 | -.194 |
| CONSP - COVID_C | -.180 | .000 | -.237 | -.123 |
| CONSP - COVID_W | -.096 | .001 | -.154 | -.037 |
| DERS_NA - DERS_G | .459 | .000 | .411 | .504 |
| DERS_NA - DERS_I | .493 | .000 | .446 | .536 |
| DERS_NA - DERS_C | .464 | .000 | .416 | .509 |
| DERS_NA - DERS_AW | .166 | .000 | .108 | .223 |
| DERS_NA - PSS | .411 | .000 | .361 | .459 |
| DERS_NA - POS_A | -.165 | .000 | -.222 | -.107 |
| DERS_NA - NEG_A | .359 | .000 | .306 | .409 |
| DERS_NA - BACQ_AP | -.162 | .000 | -.219 | -.104 |
| DERS_NA - BACQ_AV | .336 | .000 | .283 | .388 |
| DERS_NA - DSQ_AO | .194 | .000 | .137 | .251 |
| DERS_NA - DSQ_H | -.024 | .423 | -.083 | .035 |
| DERS_NA - DSQ_AN | -.080 | .008 | -.138 | -.021 |
| DERS_NA - DSQ_I | .198 | .000 | .141 | .254 |
| DERS_NA - DSQ_DEN | .112 | .000 | .053 | .170 |
| DERS_NA - DSQ_DISS | -.035 | .248 | -.094 | .024 |
| DERS_NA - DSQ_P | .350 | .000 | .297 | .400 |
| DERS_NA - DISC | .143 | .000 | .084 | .200 |
| DERS_NA - POW | .121 | .000 | .062 | .179 |
| DERS_NA - AMAC_N | .095 | .002 | .036 | .153 |
| DERS_NA - AMAC_P | -.001 | .964 | -.060 | .058 |
| DERS_NA - FUTURE | -.070 | .020 | -.129 | -.011 |
| DERS_NA - HEDONIST | .101 | .001 | .042 | .159 |
| DERS_NA - FATALIST | .181 | .000 | .123 | .237 |
| DERS_NA - COVID_S | .000 | .988 | -.060 | .059 |
| DERS_NA - COVID_C | .017 | .564 | -.042 | .076 |
| DERS_NA - COVID_W | .015 | .625 | -.044 | .074 |
| DERS_G - DERS_I | .545 | .000 | .502 | .585 |
| DERS_G - DERS_C | .358 | .000 | .305 | .408 |
| DERS_G - DERS_AW | .001 | .966 | -.058 | .060 |
| DERS_G - PSS | .523 | .000 | .479 | .565 |
| DERS_G - POS_A | -.233 | .000 | -.288 | -.176 |
| DERS_G - NEG_A | .386 | .000 | .334 | .435 |
| DERS_G - BACQ_AP | -.167 | .000 | -.224 | -.109 |
| DERS_G - BACQ_AV | .236 | .000 | .179 | .291 |
| DERS_G - DSQ_AO | .301 | .000 | .246 | .353 |
| DERS_G - DSQ_H | -.024 | .434 | -.083 | .036 |
| DERS_G - DSQ_AN | -.106 | .000 | -.164 | -.047 |
| DERS_G - DSQ_I | .132 | .000 | .074 | .190 |
| DERS_G - DSQ_DEN | -.006 | .849 | -.065 | .053 |
| DERS_G - DSQ_DISS | -.153 | .000 | -.210 | -.094 |
| DERS_G - DSQ_P | .314 | .000 | .259 | .366 |
| DERS_G - DISC | .185 | .000 | .127 | .241 |
| DERS_G - POW | .097 | .001 | .038 | .155 |
| DERS_G - AMAC_N | .050 | .100 | -.010 | .108 |
| DERS_G - AMAC_P | .002 | .953 | -.057 | .061 |
| DERS_G - FUTURE | -.203 | .000 | -.258 | -.145 |
| DERS_G - HEDONIST | .136 | .000 | .078 | .194 |
| DERS_G - FATALIST | .241 | .000 | .185 | .296 |
| DERS_G - COVID_S | -.004 | .904 | -.063 | .055 |
| DERS_G - COVID_C | .016 | .589 | -.043 | .075 |
| DERS_G - COVID_W | .066 | .029 | .007 | .124 |
| DERS_I - DERS_C | .373 | .000 | .321 | .423 |
| DERS_I - DERS_AW | .070 | .021 | .011 | .128 |
| DERS_I - PSS | .405 | .000 | .354 | .453 |
| DERS_I - POS_A | -.089 | .003 | -.147 | -.030 |
| DERS_I - NEG_A | .325 | .000 | .271 | .377 |
| DERS_I - BACQ_AP | -.146 | .000 | -.204 | -.088 |
| DERS_I - BACQ_AV | .268 | .000 | .213 | .322 |
| DERS_I - DSQ_AO | .485 | .000 | .439 | .529 |
| DERS_I - DSQ_H | -.049 | .101 | -.108 | .010 |
| DERS_I - DSQ_AN | -.089 | .003 | -.147 | -.030 |
| DERS_I - DSQ_I | .180 | .000 | .122 | .237 |
| DERS_I - DSQ_DEN | .151 | .000 | .093 | .208 |
| DERS_I - DSQ_DISS | .011 | .715 | -.048 | .070 |
| DERS_I - DSQ_P | .345 | .000 | .292 | .396 |
| DERS_I - DISC | .096 | .001 | .037 | .154 |
| DERS_I - POW | .122 | .000 | .063 | .180 |
| DERS_I - AMAC_N | .106 | .000 | .047 | .164 |
| DERS_I - AMAC_P | -.060 | .045 | -.119 | -.001 |
| DERS_I - FUTURE | -.139 | .000 | -.197 | -.081 |
| DERS_I - HEDONIST | .203 | .000 | .145 | .259 |
| DERS_I - FATALIST | .358 | .000 | .305 | .408 |
| DERS_I - COVID_S | -.031 | .306 | -.090 | .028 |
| DERS_I - COVID_C | .008 | .785 | -.051 | .067 |
| DERS_I - COVID_W | -.012 | .679 | -.072 | .047 |
| DERS_C - DERS_AW | .295 | .000 | .240 | .348 |
| DERS_C - PSS | .412 | .000 | .361 | .459 |
| DERS_C - POS_A | -.230 | .000 | -.285 | -.173 |
| DERS_C - NEG_A | .355 | .000 | .302 | .405 |
| DERS_C - BACQ_AP | -.269 | .000 | -.323 | -.213 |
| DERS_C - BACQ_AV | .371 | .000 | .318 | .420 |
| DERS_C - DSQ_AO | .236 | .000 | .179 | .291 |
| DERS_C - DSQ_H | -.050 | .095 | -.109 | .009 |
| DERS_C - DSQ_AN | -.110 | .000 | -.168 | -.051 |
| DERS_C - DSQ_I | .331 | .000 | .278 | .383 |
| DERS_C - DSQ_DEN | .183 | .000 | .125 | .239 |
| DERS_C - DSQ_DISS | .026 | .396 | -.034 | .085 |
| DERS_C - DSQ_P | .415 | .000 | .365 | .463 |
| DERS_C - DISC | .142 | .000 | .084 | .200 |
| DERS_C - POW | .169 | .000 | .111 | .225 |
| DERS_C - AMAC_N | .074 | .013 | .015 | .133 |
| DERS_C - AMAC_P | -.027 | .368 | -.086 | .032 |
| DERS_C - FUTURE | -.220 | .000 | -.275 | -.163 |
| DERS_C - HEDONIST | .152 | .000 | .094 | .210 |
| DERS_C - FATALIST | .242 | .000 | .186 | .297 |
| DERS_C - COVID_S | -.026 | .387 | -.085 | .033 |
| DERS_C - COVID_C | .007 | .808 | -.052 | .066 |
| DERS_C - COVID_W | -.003 | .911 | -.062 | .056 |
| DERS_AW - PSS | .188 | .000 | .130 | .244 |
| DERS_AW - POS_A | -.128 | .000 | -.186 | -.069 |
| DERS_AW - NEG_A | .124 | .000 | .065 | .182 |
| DERS_AW - BACQ_AP | -.378 | .000 | -.427 | -.326 |
| DERS_AW - BACQ_AV | .194 | .000 | .137 | .250 |
| DERS_AW - DSQ_AO | .009 | .771 | -.050 | .068 |
| DERS_AW - DSQ_H | -.121 | .000 | -.179 | -.062 |
| DERS_AW - DSQ_AN | -.180 | .000 | -.237 | -.122 |
| DERS_AW - DSQ_I | .219 | .000 | .161 | .274 |
| DERS_AW - DSQ_DEN | .118 | .000 | .059 | .176 |
| DERS_AW - DSQ_DISS | .003 | .910 | -.056 | .062 |
| DERS_AW - DSQ_P | .242 | .000 | .185 | .297 |
| DERS_AW - DISC | .018 | .544 | -.041 | .077 |
| DERS_AW - POW | .159 | .000 | .101 | .216 |
| DERS_AW - AMAC_N | .025 | .404 | -.034 | .084 |
| DERS_AW - AMAC_P | -.025 | .410 | -.084 | .034 |
| DERS_AW - FUTURE | -.190 | .000 | -.246 | -.132 |
| DERS_AW - HEDONIST | -.003 | .917 | -.062 | .056 |
| DERS_AW - FATALIST | .093 | .002 | .035 | .152 |
| DERS_AW - COVID_S | .011 | .726 | -.049 | .070 |
| DERS_AW - COVID_C | .010 | .745 | -.049 | .069 |
| DERS_AW - COVID_W | -.033 | .269 | -.092 | .026 |
| PSS - POS_A | -.386 | .000 | -.435 | -.334 |
| PSS - NEG_A | .541 | .000 | .497 | .581 |
| PSS - BACQ_AP | -.317 | .000 | -.369 | -.263 |
| PSS - BACQ_AV | .281 | .000 | .225 | .334 |
| PSS - DSQ_AO | .360 | .000 | .307 | .410 |
| PSS - DSQ_H | -.086 | .004 | -.145 | -.027 |
| PSS - DSQ_AN | -.206 | .000 | -.261 | -.148 |
| PSS - DSQ_I | .142 | .000 | .083 | .199 |
| PSS - DSQ_DEN | -.077 | .011 | -.135 | -.018 |
| PSS - DSQ_DISS | -.308 | .000 | -.360 | -.253 |
| PSS - DSQ_P | .465 | .000 | .418 | .510 |
| PSS - DISC | .170 | .000 | .112 | .227 |
| PSS - POW | .133 | .000 | .075 | .191 |
| PSS - AMAC_N | -.016 | .594 | -.075 | .043 |
| PSS - AMAC_P | .049 | .103 | -.010 | .108 |
| PSS - FUTURE | -.247 | .000 | -.301 | -.190 |
| PSS - HEDONIST | .056 | .061 | -.003 | .115 |
| PSS - FATALIST | .216 | .000 | .159 | .272 |
| PSS - COVID_S | .070 | .019 | .011 | .129 |
| PSS - COVID_C | .072 | .017 | .013 | .130 |
| PSS - COVID_W | .114 | .000 | .055 | .171 |
| POS_A - NEG_A | -.419 | .000 | -.467 | -.369 |
| POS_A - BACQ_AP | .262 | .000 | .206 | .316 |
| POS_A - BACQ_AV | -.177 | .000 | -.233 | -.119 |
| POS_A - DSQ_AO | -.025 | .411 | -.084 | .034 |
| POS_A - DSQ_H | .174 | .000 | .116 | .231 |
| POS_A - DSQ_AN | .153 | .000 | .095 | .210 |
| POS_A - DSQ_I | -.086 | .004 | -.145 | -.027 |
| POS_A - DSQ_DEN | .116 | .000 | .057 | .174 |
| POS_A - DSQ_DISS | .248 | .000 | .191 | .302 |
| POS_A - DSQ_P | -.233 | .000 | -.288 | -.176 |
| POS_A - DISC | -.107 | .000 | -.165 | -.048 |
| POS_A - POW | -.099 | .001 | -.158 | -.041 |
| POS_A - AMAC_N | .016 | .600 | -.043 | .075 |
| POS_A - AMAC_P | .048 | .109 | -.011 | .107 |
| POS_A - FUTURE | .178 | .000 | .120 | .234 |
| POS_A - HEDONIST | .042 | .165 | -.017 | .101 |
| POS_A - FATALIST | -.001 | .984 | -.060 | .058 |
| POS_A - COVID_S | -.016 | .604 | -.075 | .043 |
| POS_A - COVID_C | -.045 | .137 | -.104 | .014 |
| POS_A - COVID_W | -.030 | .320 | -.089 | .029 |
| NEG_A - BACQ_AP | -.236 | .000 | -.291 | -.180 |
| NEG_A - BACQ_AV | .279 | .000 | .223 | .332 |
| NEG_A - DSQ_AO | .236 | .000 | .179 | .291 |
| NEG_A - DSQ_H | -.072 | .017 | -.130 | -.013 |
| NEG_A - DSQ_AN | -.165 | .000 | -.222 | -.107 |
| NEG_A - DSQ_I | .120 | .000 | .061 | .178 |
| NEG_A - DSQ_DEN | -.051 | .093 | -.109 | .008 |
| NEG_A - DSQ_DISS | -.158 | .000 | -.215 | -.100 |
| NEG_A - DSQ_P | .370 | .000 | .318 | .420 |
| NEG_A - DISC | .211 | .000 | .153 | .266 |
| NEG_A - POW | .072 | .016 | .013 | .131 |
| NEG_A - AMAC_N | -.016 | .601 | -.075 | .043 |
| NEG_A - AMAC_P | .030 | .326 | -.029 | .089 |
| NEG_A - FUTURE | -.135 | .000 | -.193 | -.077 |
| NEG_A - HEDONIST | .101 | .001 | .042 | .159 |
| NEG_A - FATALIST | .159 | .000 | .101 | .216 |
| NEG_A - COVID_S | .051 | .089 | -.008 | .110 |
| NEG_A - COVID_C | .048 | .110 | -.011 | .107 |
| NEG_A - COVID_W | .109 | .000 | .051 | .167 |
| BACQ_AP - BACQ_AV | -.202 | .000 | -.258 | -.145 |
| BACQ_AP - DSQ_AO | -.030 | .326 | -.089 | .029 |
| BACQ_AP - DSQ_H | .126 | .000 | .068 | .184 |
| BACQ_AP - DSQ_AN | .255 | .000 | .198 | .309 |
| BACQ_AP - DSQ_I | -.255 | .000 | -.309 | -.199 |
| BACQ_AP - DSQ_DEN | -.097 | .001 | -.155 | -.038 |
| BACQ_AP - DSQ_DISS | .103 | .001 | .044 | .161 |
| BACQ_AP - DSQ_P | -.271 | .000 | -.325 | -.216 |
| BACQ_AP - DISC | .047 | .119 | -.012 | .106 |
| BACQ_AP - POW | -.133 | .000 | -.191 | -.075 |
| BACQ_AP - AMAC_N | -.041 | .170 | -.100 | .018 |
| BACQ_AP - AMAC_P | .118 | .000 | .059 | .175 |
| BACQ_AP - FUTURE | .357 | .000 | .304 | .407 |
| BACQ_AP - HEDONIST | .018 | .551 | -.041 | .077 |
| BACQ_AP - FATALIST | -.103 | .001 | -.161 | -.044 |
| BACQ_AP - COVID_S | .001 | .961 | -.058 | .061 |
| BACQ_AP - COVID_C | .004 | .890 | -.055 | .063 |
| BACQ_AP - COVID_W | .008 | .797 | -.051 | .067 |
| BACQ_AV - DSQ_AO | .194 | .000 | .136 | .250 |
| BACQ_AV - DSQ_H | -.092 | .002 | -.150 | -.033 |
| BACQ_AV - DSQ_AN | -.145 | .000 | -.202 | -.087 |
| BACQ_AV - DSQ_I | .184 | .000 | .126 | .241 |
| BACQ_AV - DSQ_DEN | .167 | .000 | .109 | .224 |
| BACQ_AV - DSQ_DISS | .037 | .220 | -.022 | .096 |
| BACQ_AV - DSQ_P | .374 | .000 | .322 | .423 |
| BACQ_AV - DISC | .073 | .015 | .014 | .131 |
| BACQ_AV - POW | .186 | .000 | .128 | .242 |
| BACQ_AV - AMAC_N | .050 | .095 | -.009 | .109 |
| BACQ_AV - AMAC_P | .018 | .540 | -.041 | .077 |
| BACQ_AV - FUTURE | -.176 | .000 | -.232 | -.118 |
| BACQ_AV - HEDONIST | .125 | .000 | .067 | .183 |
| BACQ_AV - FATALIST | .259 | .000 | .203 | .314 |
| BACQ_AV - COVID_S | -.070 | .020 | -.128 | -.011 |
| BACQ_AV - COVID_C | .013 | .664 | -.046 | .072 |
| BACQ_AV - COVID_W | .004 | .896 | -.055 | .063 |
| DSQ_AO - DSQ_H | -.012 | .680 | -.071 | .047 |
| DSQ_AO - DSQ_AN | -.041 | .176 | -.100 | .018 |
| DSQ_AO - DSQ_I | .119 | .000 | .061 | .177 |
| DSQ_AO - DSQ_DEN | .090 | .003 | .031 | .148 |
| DSQ_AO - DSQ_DISS | -.017 | .568 | -.076 | .042 |
| DSQ_AO - DSQ_P | .264 | .000 | .208 | .318 |
| DSQ_AO - DISC | .105 | .001 | .046 | .163 |
| DSQ_AO - POW | .119 | .000 | .060 | .176 |
| DSQ_AO - AMAC_N | .016 | .587 | -.043 | .075 |
| DSQ_AO - AMAC_P | .028 | .351 | -.031 | .087 |
| DSQ_AO - FUTURE | -.122 | .000 | -.180 | -.063 |
| DSQ_AO - HEDONIST | .155 | .000 | .097 | .212 |
| DSQ_AO - FATALIST | .466 | .000 | .419 | .511 |
| DSQ_AO - COVID_S | -.001 | .968 | -.060 | .058 |
| DSQ_AO - COVID_C | .011 | .726 | -.049 | .070 |
| DSQ_AO - COVID_W | .005 | .865 | -.054 | .064 |
| DSQ_H - DSQ_AN | .303 | .000 | .248 | .355 |
| DSQ_H - DSQ_I | .098 | .001 | .039 | .156 |
| DSQ_H - DSQ_DEN | .137 | .000 | .078 | .194 |
| DSQ_H - DSQ_DISS | .152 | .000 | .094 | .209 |
| DSQ_H - DSQ_P | -.107 | .000 | -.165 | -.049 |
| DSQ_H - DISC | .009 | .767 | -.050 | .068 |
| DSQ_H - POW | -.039 | .193 | -.098 | .020 |
| DSQ_H - AMAC_N | -.009 | .766 | -.068 | .050 |
| DSQ_H - AMAC_P | -.033 | .278 | -.092 | .026 |
| DSQ_H - FUTURE | .011 | .711 | -.048 | .070 |
| DSQ_H - HEDONIST | .127 | .000 | .068 | .185 |
| DSQ_H - FATALIST | -.009 | .755 | -.068 | .050 |
| DSQ_H - COVID_S | -.012 | .699 | -.071 | .047 |
| DSQ_H - COVID_C | -.002 | .947 | -.061 | .057 |
| DSQ_H - COVID_W | -.089 | .003 | -.148 | -.031 |
| DSQ_AN - DSQ_I | .086 | .004 | .027 | .144 |
| DSQ_AN - DSQ_DEN | .083 | .006 | .024 | .141 |
| DSQ_AN - DSQ_DISS | .167 | .000 | .109 | .224 |
| DSQ_AN - DSQ_P | -.144 | .000 | -.201 | -.085 |
| DSQ_AN - DISC | -.031 | .310 | -.090 | .028 |
| DSQ_AN - POW | -.052 | .082 | -.111 | .007 |
| DSQ_AN - AMAC_N | .005 | .860 | -.054 | .064 |
| DSQ_AN - AMAC_P | .012 | .695 | -.047 | .071 |
| DSQ_AN - FUTURE | .320 | .000 | .265 | .372 |
| DSQ_AN - HEDONIST | .001 | .981 | -.058 | .060 |
| DSQ_AN - FATALIST | -.140 | .000 | -.197 | -.081 |
| DSQ_AN - COVID_S | .018 | .554 | -.041 | .077 |
| DSQ_AN - COVID_C | .039 | .201 | -.021 | .097 |
| DSQ_AN - COVID_W | -.014 | .642 | -.073 | .045 |
| DSQ_I - DSQ_DEN | .532 | .000 | .489 | .573 |
| DSQ_I - DSQ_DISS | .186 | .000 | .128 | .242 |
| DSQ_I - DSQ_P | .230 | .000 | .173 | .285 |
| DSQ_I - DISC | .018 | .547 | -.041 | .077 |
| DSQ_I - POW | .146 | .000 | .087 | .203 |
| DSQ_I - AMAC_N | .066 | .028 | .007 | .125 |
| DSQ_I - AMAC_P | -.078 | .009 | -.137 | -.019 |
| DSQ_I - FUTURE | -.149 | .000 | -.207 | -.091 |
| DSQ_I - HEDONIST | .139 | .000 | .080 | .196 |
| DSQ_I - FATALIST | .084 | .005 | .025 | .143 |
| DSQ_I - COVID_S | -.060 | .046 | -.119 | -.001 |
| DSQ_I - COVID_C | .002 | .955 | -.057 | .061 |
| DSQ_I - COVID_W | -.086 | .004 | -.144 | -.027 |
| DSQ_DEN - DSQ_DISS | .520 | .000 | .476 | .562 |
| DSQ_DEN - DSQ_P | .152 | .000 | .094 | .209 |
| DSQ_DEN - DISC | -.028 | .359 | -.087 | .031 |
| DSQ_DEN - POW | .134 | .000 | .076 | .192 |
| DSQ_DEN - AMAC_N | .167 | .000 | .109 | .224 |
| DSQ_DEN - AMAC_P | -.138 | .000 | -.195 | -.079 |
| DSQ_DEN - FUTURE | -.077 | .011 | -.135 | -.018 |
| DSQ_DEN - HEDONIST | .189 | .000 | .131 | .245 |
| DSQ_DEN - FATALIST | .113 | .000 | .054 | .171 |
| DSQ_DEN - COVID_S | -.124 | .000 | -.182 | -.066 |
| DSQ_DEN - COVID_C | -.076 | .011 | -.135 | -.017 |
| DSQ_DEN - COVID_W | -.202 | .000 | -.258 | -.145 |
| DSQ_DISS - DSQ_P | .003 | .921 | -.056 | .062 |
| DSQ_DISS - DISC | -.138 | .000 | -.195 | -.079 |
| DSQ_DISS - POW | .087 | .004 | .028 | .145 |
| DSQ_DISS - AMAC_N | .134 | .000 | .075 | .191 |
| DSQ_DISS - AMAC_P | -.122 | .000 | -.180 | -.064 |
| DSQ_DISS - FUTURE | .041 | .176 | -.018 | .100 |
| DSQ_DISS - HEDONIST | .188 | .000 | .130 | .244 |
| DSQ_DISS - FATALIST | .032 | .282 | -.027 | .091 |
| DSQ_DISS - COVID_S | -.150 | .000 | -.207 | -.092 |
| DSQ_DISS - COVID_C | -.123 | .000 | -.181 | -.065 |
| DSQ_DISS - COVID_W | -.217 | .000 | -.273 | -.160 |
| DSQ_P - DISC | .065 | .032 | .006 | .123 |
| DSQ_P - POW | .221 | .000 | .164 | .276 |
| DSQ_P - AMAC_N | .108 | .000 | .049 | .166 |
| DSQ_P - AMAC_P | -.091 | .002 | -.150 | -.032 |
| DSQ_P - FUTURE | -.209 | .000 | -.265 | -.152 |
| DSQ_P - HEDONIST | .124 | .000 | .065 | .181 |
| DSQ_P - FATALIST | .196 | .000 | .138 | .252 |
| DSQ_P - COVID_S | -.057 | .059 | -.116 | .002 |
| DSQ_P - COVID_C | -.040 | .187 | -.099 | .019 |
| DSQ_P - COVID_W | -.037 | .217 | -.096 | .022 |
| DISC - POW | .109 | .000 | .051 | .167 |
| DISC - AMAC_N | .088 | .004 | .029 | .146 |
| DISC - AMAC_P | .010 | .746 | -.049 | .069 |
| DISC - FUTURE | -.002 | .949 | -.061 | .057 |
| DISC - HEDONIST | .204 | .000 | .146 | .259 |
| DISC - FATALIST | .138 | .000 | .080 | .196 |
| DISC - COVID_S | -.024 | .428 | -.083 | .035 |
| DISC - COVID_C | .044 | .142 | -.015 | .103 |
| DISC - COVID_W | .071 | .019 | .012 | .129 |
| POW - AMAC_N | .245 | .000 | .189 | .300 |
| POW - AMAC_P | -.131 | .000 | -.188 | -.072 |
| POW - FUTURE | -.114 | .000 | -.172 | -.055 |
| POW - HEDONIST | .138 | .000 | .080 | .196 |
| POW - FATALIST | .174 | .000 | .116 | .230 |
| POW - COVID_S | -.179 | .000 | -.235 | -.121 |
| POW - COVID_C | -.069 | .023 | -.127 | -.009 |
| POW - COVID_W | -.106 | .000 | -.164 | -.047 |
| AMAC_N - AMAC_P | -.411 | .000 | -.458 | -.360 |
| AMAC_N - FUTURE | .016 | .592 | -.043 | .075 |
| AMAC_N - HEDONIST | .184 | .000 | .126 | .240 |
| AMAC_N - FATALIST | .161 | .000 | .102 | .218 |
| AMAC_N - COVID_S | -.376 | .000 | -.425 | -.324 |
| AMAC_N - COVID_C | -.313 | .000 | -.365 | -.259 |
| AMAC_N - COVID_W | -.304 | .000 | -.356 | -.249 |
| AMAC_P - FUTURE | .136 | .000 | .078 | .194 |
| AMAC_P - FATALIST | .039 | .202 | -.021 | .097 |
| AMAC_P - COVID_S | .296 | .000 | .241 | .349 |
| AMAC_P - COVID_C | .309 | .000 | .254 | .361 |
| AMAC_P - COVID_W | .321 | .000 | .266 | .372 |
| FUTURE - HEDONIST | -.187 | .000 | -.243 | -.129 |
| FUTURE - FATALIST | -.286 | .000 | -.339 | -.231 |
| FUTURE - COVID_S | .099 | .001 | .040 | .157 |
| FUTURE - COVID_C | .056 | .061 | -.003 | .115 |
| FUTURE - COVID_W | .075 | .013 | .016 | .134 |
| HEDONIST - FATALIST | .298 | .000 | .243 | .351 |
| HEDONIST - COVID_S | -.140 | .000 | -.198 | -.082 |
| HEDONIST - COVID_C | -.109 | .000 | -.167 | -.050 |
| HEDONIST - COVID_W | -.123 | .000 | -.181 | -.065 |
| FATALIST - COVID_S | -.070 | .019 | -.129 | -.011 |
| FATALIST - COVID_C | .003 | .913 | -.056 | .062 |
| FATALIST - COVID_W | -.010 | .750 | -.069 | .049 |
| COVID_S - COVID_C | .384 | .000 | .332 | .433 |
| COVID_S - COVID_W | .344 | .000 | .291 | .395 |
| COVID_C - COVID_W | .294 | .000 | .239 | .347 |

a. Estimation is based on Fisher's r-to-z transformation with bias adjustment.
